# Association of physical activity with psychological distress and happiness in mothers of children with autism spectrum disorders during the COVID-19 pandemic

**DOI:** 10.1186/s12905-023-02597-5

**Published:** 2023-08-27

**Authors:** Kenji Tsunoda, Kai Nagase, Kumi Fujita

**Affiliations:** Faculty of Social Welfare, Yamaguchi Prefectural University, 6-2-1 Sakurabatake, 753-0021 Yamaguchi, Yamaguchi Japan

**Keywords:** Exercise, Developmental disorders, Parents, Mental health, Well-being

## Abstract

**Background:**

The coronavirus disease 2019 (COVID-19) pandemic may have severely impacted the psychological status of mothers of children with autism spectrum disorder (ASD). Although a previous study reported that physical activity (PA) moderated psychological distress in parents of children with ASD during the COVID-19 pandemic, the effect of PA on the happiness levels of such parents during the pandemic remains unclear. This study investigated the associations among PA, psychological distress, and happiness in mothers of children with ASD during the COVID-19 pandemic.

**Methods:**

This cross-sectional study was designed to evaluate mothers of children with disabilities. Questionnaires were collected from mothers living in Yamaguchi and Okayama Prefectures, Japan, between February and December 2022. During this period, three large waves of the COVID-19 pandemic occurred in Japan. Of the 601 respondents, 334 mothers had children with ASD and offered valid data. PA was assessed using the short form of the International Physical Activity Questionnaire. Psychological distress and happiness were assessed using the six-item Kessler Psychological Distress Scale (K6) and the Subjective Happiness Scale (SHS).

**Results:**

The mothers had markedly higher K6 scores (6.49) and more than half of them had moderate-to-severe psychological distress, whereas the SHS scores (4.46) were similar to that of the general Japanese population. In a multivariable-adjusted model based on the analysis of covariance, the K6 score was not associated with any PA items. In contrast, SHS scores were positively associated with moderate-intensity PA (MPA) and total moderate- to vigorous-intensity PA (MVPA), independent of K6. In the post-hoc test, mothers who did some (4.52) or enough (≥ 150 min/week) MPA (4.56) had a higher SHS score than those who did not (4.09). Similarly, mothers who engaged in sufficient (≥ 600 MET-min/week) total MVPA had higher SHS scores (4.57) than those who did not engage in MVPA (4.12).

**Conclusions:**

The mothers of children with ASD during the COVID-19 pandemic had markedly higher psychological distress, though none of the PA items were associated with stress levels. However, PA was positively associated with happiness in mothers of children with ASD independent of their stress levels.

## Background

The parents of children with autism spectrum disorder (ASD) generally experience higher stress compared with parents of children displaying typical development or those diagnosed with other disabilities, including Down syndrome, intellectual disability, and cerebral palsy [1]. For example, known traits of ASD such as impaired social communication and restricted or repetitive behaviors can be a cause of severe stress for parents [1–3]. Although the coronavirus disease 2019 (COVID-19) pandemic had a substantial negative impact on the mental health of people around the world [4], this effect may have been more severe in parents of children with ASD than in other populations due to the difficulty in attending school and support institutions increasing their burden [5–7]. Women are more likely to experience depressive symptoms than men [8], and the prevalence of depressive and anxiety disorders displayed a greater increase in women than in men during the COVID-19 pandemic [4, 9]. Additionally, a recent longitudinal study indicated that, during the COVID-19 pandemic, mothers of children with ASD reported higher levels of psychological distress compared to fathers [10]. As a parent’s negative psychological status is linked to psychological and behavioral problems in their children [11], identifying the correlates of psychological status among mothers of children with ASD is important for supporting both their own well-being and that of their children.

Depression and anxiety represent negative psychological constructs, and there are some studies that explore preventing or moderating them among the parents of children with ASD [12, 13]. Happiness is recognized as a positive psychological construct, and some studies examined happiness among parents of children with ASD [14, 15]. A recent Polish study reported that parents of children with ASD reported lower levels of happiness and higher levels of depression than parents of neurotypical children [14]. There has been a growing belief that we should focus not only on preventing negative situations, such as depression, but also focus on positive aspects of life such as happiness [16]. Although parents of children with ASD generally face difficult psychological circumstances, some experience happiness despite difficult situations [17]. However, the factors that promote or maintain happiness in parents of children with ASD remain unclear.

Physical activity (PA) is a known factor in maintaining better psychological status, and its beneficial effect on the prevention of depression is well-known [18]. A recent Chinese study delivered a web-based parent-child PA program to parents of children with ASD during the COVID-19 pandemic and confirmed a noticeable reduction in stress, anxiety, and depression compared to the control group [13]. Although this study revealed the alleviating effect the program had on negative psychological constructs, it did not focus on positive constructs, and the effect of PA on happiness remains unclear. As a positive association between PA and happiness has been reported in the general population [19], it may be observed in the parents of children with ASD as well. A positive association between PA and happiness among mothers of children with ASD could help develop methods for managing the psychological well-being of mothers of children with ASD.

Thus, this study aimed to investigate the association of PA with psychological distress and happiness in mothers of children with ASD during the COVID-19 pandemic.

## Methods

### Study design and participants

This cross-sectional study included the mothers of children with disabilities. Questionnaires were sent to special schools, after-school daycare centers, child developmental support centers, and associations with friends of parents of children with disabilities in Yamaguchi and Okayama Prefectures, Japan between February and December, 2022. Three large waves of the COVID-19 pandemic occurred in Japan during the survey period [20]. Responses to the questionnaire were provided an anonymous form, and in addition to paper forms (n = 527), respondents were given the option to respond via the Internet (Google Forms) (n = 74). Of the 601 respondents, 594 mothers provided responses on the types of disabilities their children were diagnosed with, and 352 of them had children diagnosed with ASD. Of these, cases in which the child was an adult (≥ 20 years old) (n = 14) and cases missing data on age of the child (n = 4) were excluded. In total, data from 334 mothers were included in the analysis. The mean (standard deviation [SD]) age of the mothers was 43.7 (5.5) years and the mean age of their children with ASD was 11.9 (3.9) years old. For data with missing values (n = 94; 28.1%), multiple imputations were performed to avoid the loss of information.

All data were collected using an anonymous self-report questionnaire. The survey’s purpose and data used for research were explained in a consent cover letter, and informed consent was obtained by the voluntary return of the questionnaires. All methods were carried out in accordance with the relevant guidelines and regulations. The Ethics Committee of Yamaguchi Prefectural University approved the study protocol (Ref. No. 2021-33).

### Measurements of variables

#### Psychological distress

Psychological distress was assessed as an indicator of negative psychological status using the Japanese version of the six-item Kessler Psychological Distress Scale (K6) [21]. The six items were rated on a scale of 0 to 4 points, with the total being the K6 score. The total K6 score ranged from 0 to 24. A score ≥ 5 on the K6 was considered to indicate moderate psychological distress, and a score ≥ 13 was considered severe [22].

#### Happiness

The Subjective Happiness Scale (SHS) was also used [23, 24] in this study. The SHS comprises four questions, each rated on a Likert scale ranging from 1 to 7. The average score of the four items was used for the overall evaluation. Item 1 asks respondents to characterize themselves using absolute ratings, where “In general, I consider myself:” with the respondent being “not a very happy person” to “a very happy person.” Item 2 also asks respondents to characterize themselves, but it uses relative ratings to peers, where “Compared to most of my peers, I consider myself:” with the respondent being “less happy” to “more happy. Items 3 and 4 offer brief descriptions of happy and unhappy individuals and ask respondents the extent to which each characterization describes them. Specifically, Item 3 asks “Some people are generally very happy. They enjoy life regardless of what is going on and get the most out of everything. To what extent does this characterization describe you?” and Item 4 asks “Some people are generally not very happy. Although they are not depressed, they never seem as happy as they might be. To what extent does this characterization describe you?” Responses to both Item 3 and Item 4 range from “not at all” to “a great deal,” but Item 4 is reverse-scored. This study used each item score of the SHS for analysis, in addition to the overall evaluation.

#### Physical activity

PA levels were assessed using the International Physical Activity Questionnaire (IPAQ) short form [25]. Participants were asked to report the frequency and duration of three types of PA performed in bouts of ≥ 10 min in a usual week, including walking corresponding to 3.3 MET, moderate-intensity PA (MPA) (e.g., carrying light loads, swimming at a slow pace, or playing doubles tennis) corresponding to 4.0 MET, and vigorous-intensity PA (VPA) (e.g., carrying heavy loads, running, cycling up a hill, or playing single tennis) corresponding to 8.0 MET. As the intensity of walking is coded as 3.3 MET in the IPAQ short form, which is greater than the intensity of widely accepted definitions of MPA (≥ 3.0) [26], we incorporated walking into MPA in the calculation of PA volume. Total moderate- to vigorous-intensity PA (MVPA) was calculated as the sum of these types of PA. Based on the PA volume recommended by the World Health Organization (WHO) [27, 28], MPA (0, ≤ 149, ≥150 min/week), VPA (0, ≤ 74, ≥75 min/week), and total MVPA (0, ≤ 599, ≥600 MET-minutes/week) were divided into three categories. The highest level of each PA item corresponds to the PA level recommended by the WHO.

#### Other variables

Demographic variables included age, number of children with ASD (one or more), working status (unemployed, non-regular employment, or regular employment), subjective economic status (good, slightly good, slightly poor, or poor), and living arrangements (yes or no) with people other than the child’s mother (fathers, siblings, or grandparents).

### Statistical analysis

Among all the participants, 94 (28.1%) had missing data for at least one item. Multiple imputations were performed to maintain the sample balance. We created 20 multiple imputed datasets, analyzed them separately, and pooled the results [29].

To check basic information, we examined the correlations between psychological distress and happiness using crude (Pearson’s r) and adjusted models (partial r). The adjusted model included age, number of children with ASD, subjective economic status, work status, and living arrangements.

To compare psychological status according to PA level, we used an analysis of covariance. If there was a significant linear trend, we conducted a post-hoc test with the Bonferroni correction. We developed two multivariable-adjusted models. Model 1 included age, number of children with ASD, subjective economic status, working status, living arrangements, and another type of PA intensity (VPA/MPA) in the MPA/VPA analysis. Model 2 added the K6/SHS to Model 1 in relation to one another. All statistical analyses were conducted using SPSS version 29.0.1 (IBM Corp., Armonk, NY, USA), and the level of significance was set at *P* < 0.05.

## Results

Descriptive statistics are shown in Table 1. The mean (SD) K6 score was 6.49 (5.28), and more than half of the participants had moderate (43.8%) to severe psychological distress (12.5%). The mean SHS score was 4.46 (1.27). When looking at the individual items of the SHS, Item 3, “Feeling happy in any situation,” was the lowest-scoring item (4.06 [1.62]).

**Table 1:** Characteristics of participants

	n (%)	Missing n (%)
**M (SD) age, years**	43.7 (5.5)	60 (18.0)
**Number of children with ASD**		0 (0.0)
1	290 (86.8)	
≥2	44 (13.2)	
**Subjective economic status**		3 (0.9)
Good	12 (3.6)	
Slightly good	126 (38.1)	
Slightly poor	144 (43.5)	
Poor	49 (14.8)	
**Working status**		6 (1.8)
Unemployed	102 (31.1)	
Non-regular employment	134 (40.9)	
Regular employment	92 (28.0)	
**Living arrangements for children**		
Father, yes	287 (85.9)	0 (0.0)
Siblings, yes	264 (79.0)	0 (0.0)
Grandparents, yes	59 (17.7)	0 (0.0)
**Physical activity**		
**M (SD) moderate-intensity PA, min/week**	343 (493)	25 (7.5)
0	70 (22.7)	
≤149	89 (28.8)	
≥150	150 (48.5)	
**M (SD) vigorous-intensity PA, min/week**	61 (176)	9 (2.7)
0	252 (77.5)	
≤74	27 (8.3)	
≥75	46 (14.2)	
**M (SD) total MVPA, MET-min/week**	1691 (2794)	29 (8.7)
0	68 (22.3)	
≤599	92 (30.2)	
≥600	145 (47.5)	
**M (SD) K6 score, points**	6.49 (5.28)	5 (1.5)
Low (≤ 4)	144 (43.8)	
Moderate (5–12)	144 (43.8)	
Severe (≥ 13)	41 (12.5)	
**M (SD) SHS score, points**	4.46 (1.27)	10 (3.0)
#1. Happiness in absolute ratings	4.73 (1.49)	5 (1.5)
#2. Happiness in relative ratings	4.34 (1.45)	6 (1.8)
#3. Feeling happy in any situation	4.06 (1.62)	5 (1.5)
#4. Feeling unhappy despite not being depressed†	4.70 (1.65)	8 (2.4)

Values are presented as numbers (percentages) unless stated otherwise. †Reversed score in a positive direction. ASD: autism spectrum disorders; K6: Kessler Psychological Distress Scale; M: mean; MVPA: moderate- to vigorous-intensity physical activity; PA: physical activity; SD: standard deviation; SHS: Subjective Happiness Scale

Table 2 shows the correlation between psychological distress and happiness. In the adjusted model, the SHS score was moderately correlated with the K6 score (partial r = -0.55). The lowest correlation among the individual items of the SHS with the K6 was in the reversed score of Item 4, “Feeling unhappy despite not being depressed” (partial r = -0.41).

**Table 2:** Correlations between psychological distress and happiness

	Items of the Subjective Happiness Scale				Subjective Happiness Scale score
	#1. Happiness in absolute ratings	#2. Happiness in relative ratings	#3. Feeling happy in any situation	#4. Feeling unhappy despite not being depressed†	
**K6 score**					
r	-0.53*	-0.48*	-0.46*	-0.45*	-0.59*
(r^2^)	(0.28)	(0.23)	(0.21)	(0.20)	(0.35)
Partial r‡	-0.49*	-0.44*	-0.43*	-0.41*	-0.55*
(Partial r^2^‡)	(0.24)	(0.20)	(0.18)	(0.17)	(0.31)

These values were based on multiple imputations. The significance of partial *r* was based on the average P values. *P < 0.05. †The reversed score in the positive direction was entered. ‡Adjusted for age, number of children with autism spectrum disorders, subjective economic status, working status, and living arrangements (fathers, siblings, and grandparents). K6: Kessler Psychological Distress Scale

Table 3 shows the associations between PA, psychological distress, and happiness. The K6 score was not associated with any PA variables in Models 1 or 2. In contrast, SHS score was positively associated with MPA and total MVPA in both Models. In the post-hoc test, mothers who met the WHO recommendation level in MPA, i.e. ≥150 min/week (mean [95% confidence interval] = 4.56 [4.41 to 4.71]) or lightly engaged in MPA, i.e. ≤149 min/week (4.52 [4.31 to 4.73]) had a higher SHS score than those who did not engage in MPA (4.09 [3.85 to 4.32]). Similarly, mothers who met the WHO recommendation level in total MVPA, i.e. ≥600 MET-min/week had a higher SHS score (4.57 [4.43 to 4.72]) than those who did not engage in MVPA (4.12 [3.88 to 4.35]).

**Table 3:** Comparisons of psychological distress and happiness by physical activity levels

	K6, mean (95% confidence interval)				SHS, mean (95% confidence interval)			
	Model 1		Model 2		Model 1		Model 2	
**Moderate-intensity PA (min/week)**								
0 (a)	6.93	(5.69, 8.16)	5.91	(4.87, 6.96)	4.04	(3.76, 4.32)	4.09	(3.85, 4.32)
≤149 (b)	6.40	(5.30, 7.49)	6.60	(5.67, 7.53)	4.53	(4.29, 4.78)	4.52	(4.31, 4.73)
≥150 (c)	6.39	(5.59, 7.19)	6.69	(6.02, 7.37)	4.57	(4.40, 4.75)	4.56	(4.41, 4.71)
Average *P* (range) for linear trend	0.483	(0.337, 0.639)	0.228	(0.149, 0.373)	0.002	(0.001, 0.003)	0.001	(< 0.001, 0.002)
Post-hoc test based on average *P*					b, c > a		b, c > a	
**Vigorous-intensity PA (min/week)**								
0 (a)	6.41	(5.75, 7.07)	6.33	(5.78, 6.88)	4.42	(4.26, 4.57)	4.40	(4.28, 4.53)
≤74 (b)	5.20	(3.19, 7.20)	6.20	(4.55, 7.85)	4.87	(4.42, 5.32)	4.70	(4.33, 5.07)
≥75 (c)	7.65	(6.04, 9.26)	7.51	(6.16, 8.86)	4.39	(4.03, 4.75)	4.54	(4.23, 4.84)
Average *P* (range) for linear trend	0.180	(0.082, 0.360)	0.129	(0.059, 0.299)	0.850	(0.682, 0.975)	0.455	(0.285, 0.731)
**Total MVPA (MET-min/week)**								
0 (a)	6.67	(5.43, 7.90)	5.79	(4.75, 6.83)	4.10	(3.81, 4.38)	4.12	(3.88, 4.35)
≤599 (b)	6.35	(5.28, 7.42)	6.44	(5.55, 7.34)	4.49	(4.25, 4.73)	4.47	(4.27, 4.67)
≥600 (c)	6.53	(5.73, 7.32)	6.82	(6.16, 7.49)	4.57	(4.39, 4.75)	4.57	(4.43, 4.72)
Average *P* (range) for linear trend	0.845	(0.742, 0.968)	0.105	(0.056, 0.159)	0.006	(0.003, 0.009)	0.001	(< 0.001, 0.003)
Post-hoc test based on average *P*					c > a		c > a	

These values were based on multiple imputations. Model 1 was adjusted for age, number of children with autism spectrum disorders, subjective economic status, working status, living arrangements (fathers, siblings, and grandparents), and another intensity type (vigorous/moderate) of PA for the analysis of moderate/vigorous-intensity PA. Model 2 was additionally adjusted for the K6/SHS in Model 1 to account for the correlation. Post-hoc tests were based on the Bonferroni correction. K6: Kessler Psychological Distress Scale; MVPA: moderate-to-vigorous-intensity physical activity; PA: physical activity; SHS: Subjective Happiness Scale

Table 4 shows the associations between individual items of the SHS and PA. The individual items of the SHS, except Item 2 “happiness in relative ratings”, were positively associated with MPA and these associations were independent of K6. For total MVPA, the individual items of the SHS, except Item 1 “Happiness in absolute ratings”, were positively associated.

**Table 4:** Comparisons of Subjective Happiness Scale components by physical activity levels

	Items of the Subjective Happiness Scale, mean (95% confidence interval)							
	#1. Happiness in absolute ratings		#2. Happiness in relative ratings		#3. Feeling happy in any situation		#4. Feeling unhappy despite not being depressed†	
**Moderate-intensity PA (min/week)**								
0 (a)	4.42	(4.12, 4.71)	4.07	(3.77, 4.37)	3.58	(3.25, 3.92)	4.29	(3.94, 4.63)
≤149 (b)	4.69	(4.43, 4.95)	4.37	(4.10, 4.63)	4.41	(4.11, 4.71)	4.62	(4.31, 4.92)
≥150 (c)	4.86	(4.67, 5.05)	4.40	(4.21, 4.59)	4.06	(3.85, 4.28)	4.91	(4.69, 5.13)
Average *P* (range) for linear trend	0.015	(0.008, 0.030)	0.076	(0.048, 0.139)	0.020	(0.008, 0.035)	0.003	(0.001, 0.007)
Post-hoc test based on average *P*	c > a				b > a		c > a	
**Vigorous-intensity PA (min/week)**								
0 (a)	4.71	(4.56, 4.87)	4.26	(4.10, 4.42)	3.97	(3.79, 4.15)	4.67	(4.48, 4.85)
≤74 (b)	4.94	(4.46, 5.41)	4.59	(4.11, 5.06)	4.39	(3.85, 4.92)	4.90	(4.34, 5.46)
≥75 (c)	4.63	(4.24, 5.03)	4.46	(4.07, 4.85)	4.29	(3.85, 4.73)	4.76	(4.29, 5.23)
Average *P* (range) for linear trend	0.708	(0.319, 0.918)	0.388	(0.172, 0.813)	0.207	(0.086, 0.361)	0.726	(0.338, 0.994)
**Total MVPA (MET-min/week)**								
0 (a)	4.46	(4.16, 4.76)	4.06	(3.76, 4.36)	3.59	(3.25, 3.92)	4.36	(4.01, 4.70)
≤599 (b)	4.75	(4.50, 5.01)	4.35	(4.09, 4.60)	4.26	(3.97, 4.55)	4.52	(4.22, 4.82)
≥600 (c)	4.81	(4.62, 5.00)	4.41	(4.23, 4.60)	4.13	(3.92, 4.35)	4.94	(4.72, 5.16)
Average *P* (range) for linear trend	0.059	(0.036, 0.098)	0.049	(0.029, 0.075)	0.008	(0.002, 0.013)	0.005	(0.002, 0.011)
Post-hoc test based on average *P*			Not significant		b, c > a		c > a	

These values were based on multiple imputations. The mean, 95% confidence interval, and *P* value were adjusted for age, number of children with autism spectrum disorders, subjective economic status, working status, living arrangements (fathers, siblings, and grandparents), K6, and another intensity type (vigorous/moderate) of PA for the analysis of moderate/vigorous-intensity PA. Post-hoc tests were based on the Bonferroni correction. †The reversed score in the positive direction was entered. K6: Kessler Psychological Distress Scale; MVPA: moderate-to-vigorous-intensity physical activity; PA: physical activity

## Discussion

The COVID-19 pandemic affected the well-being of various people. In a national survey in Japan conducted before the COVID-19 pandemic, the mean K6 score was 2.8 points, similar to a national survey in the United States [30]. However, significantly increased psychological distress was observed in the Japanese population during the mild lockdown caused by the COVID-19 pandemic, with the mean K6 score at 5.58 [31]. Notably, mothers of children with ASD in this study had higher K6 scores (6.49) and more than half of the respondents had moderate to severe psychological distress. Not only was the psychological burden of the pandemic more severe among women [4, 9], but social support for children with ASD in schools and support institutions was also remitted during the COVID-19 pandemic [5, 6]. Additionally, a recent study from the United States reported higher levels of psychological distress among mothers of children with ASD during the COVID-19 pandemic, compared with fathers [10], which supports the markedly higher psychological distress observed among the mothers in this study. Although some studies have reported PA as an important factor in maintaining better psychological status during the COVID-19 pandemic [32, 33], little information is available on mothers of children with ASD, who endure severe psychological distress [13]. Therefore, examining the associations of PA with psychological distress and happiness in these individuals is crucial for managing their psychological well-being.

PA has been found to alleviate psychological distress [18, 32–35]. For example, physiological studies suggest that PA can improve elevated levels of pro-inflammatory cytokines and deficits in the production of brain-derived neurotrophic factor, considered as the pathophysiology of depression [35]. Additionally, a systematic review of epidemiological studies confirmed a dose-response relationship between higher PA volumes and a lower risk of depression, and estimated that 11.5% of depression cases could have been prevented if less-active adults had met WHO recommendations for PA [18]. Despite this evidence suggesting a relationship between PA and psychological distress, no significant association was found among mothers of children with ASD in this study. However, this is not a surprising result. A meta-analysis of studies involving women with breast cancer did not confirm the effects of dance/movement therapy on depression, stress, and anxiety [36]. Breast cancer is known to cause chronic psychological distress in patients [37]. Although we do not have a definite reason for the lack of associations, PA may have been less effective in moderating the psychological status of the mothers in this study as they experienced markedly higher psychological distress over a long time due to the COVID-19 pandemic.

The SHS score was moderately correlated with the K6 score, and the coefficient of determination was 0.31, indicating that most happiness among mothers of children with ASD could be explained by factors other than psychological distress. The mean SHS score of the general Japanese population surveyed before the COVID-19 pandemic was 4.37 points [38], which is similar to the scores of mothers of children with ASD in this study (4.46 points). The results of this study suggest that although mothers of children with ASD during the COVID-19 pandemic were more likely to suffer higher psychological distress, they can still feel happy, similar to other general populations. This could be an important finding in planning promotional programs for mothers’ psychological well-being.

In association with PA, the SHS score was positively associated with MPA and total MVPA, and mothers who met the WHO recommendations for MPA or total MVPA had higher SHS scores than those who did not. These associations persisted after adjusting for K6 score. K6 was not associated with any of the PA items, which could be a reason for the independent association between the SHS score and PA items. Although a positive association between PA and happiness has been reported in the general population [19], this positive association was also found in the severely stressed population of mothers of children with ASD included in this study. Exercise has been shown to stimulate the release of endorphins and monoamines such as dopamine [39, 40], which are closely associated with happiness [41, 42]. Additionally, in a previous study, SHS score was positively associated with gray matter volume in the right precuneus [43]. PA is positively associated with gray matter volume of the brain [44]. Considering these previous studies, PA could promote positive psychological states, including happiness, through multiple beneficial effects on neural networks and the brain. In this study, PA was assessed using the IPAQ short form; although it is not clear whether this PA was derived from exercise or life-related activities, as mothers with high activity levels may enjoy exercising with their relatives and/or friends. The beneficial effects of better social relationships and happiness are well known [41, 45], and activating social interaction through exercise may enhance or maintain happiness. Future research is needed to evaluate the types of PA, such as leisure time, household-, and work-related PA, and with/without others to clarify which types of PA have a more positive impact on happiness.

In the present study, VPA alone was not associated with happiness. A recent study reported a positive association among MPA, VPA and happiness [46], which is not consistent with this finding. Although the reason for this difference is unclear, many mothers of children with ASD do not engage in VPA, which could make conducting a sound analysis challenging. In addition, because VPA in the IPAQ short form includes PA at work, mothers with higher VPA levels may engage in physically demanding jobs. Activity type, particularly whether it is exercise or not, should be examined for VPA using a sufficient sample size.

When looking at the individual items of the SHS, Items 3 and 4 were more clearly associated with MPA and total MVPA than other items. These items asked whether an individual felt happy in any situation that included aspects of psychological resilience [47]. Mothers of children with ASD had higher K6 scores, which may be a result of their exposure to adversity during the COVID-19 pandemic. As PA has protective effects against stressful circumstances [48], mothers with higher levels of PA could have higher happiness scores on these items, independent of their stress level.

This study has the advantage of using data from more than 300 individuals from a special population of mothers of children with ASD. However, it has several limitations. First, because this was a cross-sectional study, it could not demonstrate a causal relationship between PA and happiness. Although PA is thought to have a positive effect on happiness, it is possible that higher psychological resilience and happiness allow people to engage in PA. Therefore, the causal relationship between PA and happiness in mothers of children with ASD requires further investigation using prospective or interventional designs. Second, all items were assessed using a self-reported questionnaire, which may have introduced recall or reporting bias. In particular, for the assessment of PA, it is desirable to increase the validity and expand the assessment of other behaviors, including light-intensity PA (e.g., housework and gardening), through the use of objective assessments, such as an accelerometer [49, 50]. Third, although we did not assess the severity of ASD in the children of the mothers included in this study, it is a known factor for higher distress [51], and the increased severity of ASD behaviors in children due to the COVID-19 pandemic was associated with higher parental distress and lower emotional well-being [7]. Therefore, different results may be observed when the severity of ASD in children is included as a covariate or stratification factor. Finally, although mothers of children with ASD generally have higher levels of stress [1], this stress may have been even higher because this survey was conducted during the COVID-19 pandemic [5, 6]. Although this study did not observe a significant association between PA and psychological distress, it may be favorably associated under normal circumstances, as observed in other populations [18]. Additionally, the data collection for this study was restricted to Yamaguchi and Okayama prefectures, both of which lie in the Chugoku region of Japan. This region is more rural than the regions of Kanto (including Tokyo prefecture) and Kansai (including Osaka prefecture), and therefore, the generalizability of this study remains unknown. Further studies should be conducted in other areas, especially urban areas, after the end of the COVID-19 pandemic.

## Conclusions

Mothers of children with ASD during the COVID-19 pandemic had markedly higher psychological distress, though none of the PA items were associated with stress levels. However, MPA and total MVPA were associated with happiness in mothers of children with ASD, and mothers who met the WHO recommendations for MPA or total MVPA reported higher happiness than those who did not; these associations were independent of stress levels. These findings can be beneficial for managing the psychological well-being of mothers of children with ASD. Further studies, especially those with prospective and interventional designs, are needed to clarify the benefits of PA on the happiness of mothers of children with ASD.

## Data Availability

The data are not publicly available because of privacy and ethical restrictions. Data supporting the findings of this study are available from the corresponding author upon request.

## List of abbreviations

ASD: Autism spectrum disorders
COVID-19: Coronavirus disease 2019
IPAQ: International Physical Activity Questionnaire
K6: Six-item Kessler Psychological Distress Scale
MPA: Moderate-intensity physical activity
MVPA: Moderate- to vigorous-intensity physical activity
PA: Physical activity
SD: Standard deviation
SHS: Subjective Happiness Scale
VPA: Vigorous-intensity physical activity
WHO: World Health Organization
